# Beyond Bacteria: Fungi in the Tumor Microbiome

**DOI:** 10.3390/cancers15030572

**Published:** 2023-01-17

**Authors:** Kentaro Inamura

**Affiliations:** 1 Division of Pathology, The Cancer Institute, Japanese Foundation for Cancer Research, Tokyo 135-8550, Japan; kentaro.inamura@jfcr.or.jp; Tel.: +81-3-3570-0111 (ext. 5604); 2 Department of Pathology, The Cancer Institute Hospital, Japanese Foundation for Cancer Research, Tokyo 135-8550, Japan

## 1. Introduction

The microbiota is widely recognized to influence diverse biological processes, including metabolism, neurological and cardiovascular functions, the inflammatory response and immunity [1]. The disruption of the balanced composition of commensal microbes pathologically affects these functions. Moreover, the ecosystems created by the microbial community and host cells have a substantial impact on health, with an imbalance in their interactions leading to a wide range of diseases, including cancer [2,3]. Tumors harbor unique microbial communities that vary by tumor type and may persist throughout the metastatic process. Considering the critical roles played by polymorphically variable microbes in cancer initiation and progression, “polymorphic microbiomes” have recently been added to the hallmarks of cancer [4]. Over the past few decades, numerous studies have focused on bacterial species in the tumor microbiome; however, current studies have begun to address the need to expand the scope of microbiome research to incorporate other microorganisms, such as fungi, viruses and archaea. For example, a multikingdom microbial analysis of colorectal cancer (CRC) metagenomic datasets unraveled 16 microbial biomarkers (including 11 bacterial, 4 fungal and 1 archaeal features) that performed better than single-kingdom markers in identifying CRC patients [5]. Additionally, a metagenomic functional analysis revealed that bacterial–fungal interactions may promote colorectal carcinogenesis by upregulating D-arginine and D-ornithine and by stimulating the butanoate metabolism [5]. Fungi have received relatively little attention despite their potential as commensals/opportunistic pathogens that shape host immunity and infect immunocompromised individuals, including cancer patients. Further, intratumoral fungi, which reside within the tumor or immune cells, are less abundant than intratumoral bacteria (4% vs. 96%, respectively) [6]. Despite their low abundance, intratumoral fungi are potentially immunogenic and contribute to the compositions of intratumoral fungal–bacterial–immune clusters [6]. Following two recent pancancer mycobiome studies [6,7] that, together, painted a comprehensive picture of tumor-associated fungal microbiota in a range of human malignancies, the mycobiome has garnered increasing research interest in the context of cancer pathogenesis, diagnosis and therapy.

## 2. The Study by Gamal A. et al. Published in *Cancers*

In their review article [8], Gamal et al. addressed the significant role of the mycobiome in carcinogenesis in terms of (1) microbial inflammation, (2) biofilm formation and (3) fungus-derived metabolites.

(1) Microbial inflammation: During a fungal invasion, cell-wall-derived pathogen-associated molecular patterns (PAMPs) are recognized by pattern recognition receptors (PRRs), such as Toll-like receptors. The interaction between fungal PAMPs and host PRRs triggers several signaling cascades that lead to chronic inflammation. Certain fungal species are capable of translocating from the gut to the pancreas and inducing pancreatic carcinogenesis. In particular, *Malassezia* species, known to infect the scalp or skin, were found to promote pancreatic carcinogenesis by interacting with the mannose-binding lectin, thereby activating the complement cascade [9]. Furthermore, in response to the intratumoral mycobiome, pancreatic adenocarcinoma cells appeared to produce interleukin-33 (IL-33) as a chemoattractant for type 2 immune cells, which can stimulate tumor growth by secreting protumorigenic cytokines, such as IL-4, IL-5 and IL-13 [10].
(2) Biofilm formation: The bacterial–fungal biofilm acts as a barrier that protects the microbes from the host immune system and potentially exacerbates the local inflammatory response [11]. *Candida albicans* is a well-known fungal pathogen that contributes to oral carcinogenesis. *C. albicans*, *Actinomyces naeslundii* and *Streptococcus mutans* create polymicrobial biofilms, which trigger the malignant transformation of oral keratinocytes. Biofilm effluents especially modulate the adhesion of oral squamous cell carcinoma cells to the extracellular matrix and induce the production of proinflammatory cytokines, such as IL-6 and IL-8 [12].
(3) Fungus-derived metabolites: Aflatoxin B1 (AFB1), produced by *Aspergillus* species, belongs to a class of carcinogenic mycotoxins that cause the development of hepatocellular carcinoma by generating highly mutagenic DNA adducts [13].

This review also highlighted the diagnostic implications of the mycobiome in cancer patients [8]. Recent studies have shown that fungal profiling is a potential diagnostic tool for gastroenterological (e.g., colorectal, pancreatic and head and neck/oral cancers) and nongastroenterological cancers (e.g., ovarian, breast and melanoma cancers). For example, fecal fungal dysbiosis with an increased Basidiomycota:Ascomycota ratio has been observed in CRC patients [14]. Additionally, the fecal microbiota was characterized by an increased co-occurrence of fungal intrakingdom correlations, whereas the co-occurrence of bacterial–fungal correlations (e.g., Proteobacteria and Ascomycota correlations) was decreased in CRC patients. These observations indicate that synergistic intrafungal and antagonistic bacterial–fungal associations may contribute to colorectal carcinogenesis [14]. A recent pancancer mycobiome study compared the fungal population of one cancer type with thirty-one other types of cancer, and showed that cancer type-specific intratumoral and plasma mycobiomes could be robustly discriminated, indicating the existence of distinct cancer type-specific mycobiome signatures [6]. This study further demonstrated the prognostic capabilities of intratumoral and plasma mycobiomes, even in stage I disease of various cancer types [6]. Another pancancer mycobiome study revealed that the abundance of intratumoral fungi could roughly distinguish between gastrointestinal and nongastrointestinal tumors [7]. *Blastomyces*, for example, were found to be highly prevalent in lung tumor tissues [7]. This study further revealed that gastrointestinal cancers exhibited a variation in the relative abundance of *Candida albicans* and *Saccharomyces cerevisiae*, suggesting that gastrointestinal cancers could be segregated into *Candida*-associated and *Saccharomyces*-associated tumors [7]. *Candida*-dominant tumors were associated with the activation of IL-1 proinflammatory immune pathways and increased neutrophils [7]. Furthermore, *Candida*-dominant tumors were characterized by the attenuation of cell adhesion pathways and high frequencies of stage IV metastatic disease [7].

This review also discussed the impact of the mycobiome on host responses to anticancer treatments [8]. Beta-glucan, one of the most abundant polysaccharides in the fungal cell wall, is a potential immunomodulator that augments the antitumor activity of several immunotherapies by modulating innate and adaptive immune responses [15]. For example, the oral administration of yeast-derived beta-glucan delayed tumor growth by converting immunosuppressive M2-type tumor-promoting macrophages to M1-type antitumor macrophages [16]. The relationship between the mycobiome and immunotherapy response was further explored, revealing that Capnodiales and its genus, *Cladosporium*, were significantly enriched in patients with metastatic melanoma who did not respond to immune checkpoint inhibitors [6]. Next, a recent study elucidated the divergent and opposing roles of commensal fungi and bacteria in regulating tumor responses to radiation therapy [17]. In mouse models with breast cancer and melanoma, the antibiotic-mediated depletion or gnotobiotic exclusion of fungi enhanced the responsiveness to radiation, whereas antibiotic-mediated bacterial ablation reduced the responsiveness, which was associated with the overgrowth of commensal fungi [17].

In conclusion, despite increasing efforts to elucidate the complex host–tumor–microbiome interactions, research on the tumor mycobiome is still in its infancy. Further extensive research is needed to examine the relationships between cancer and various microbes, including fungi. To develop microbiome-modulating strategies for cancer control, we should gain insights into the complex and dynamic relationships between cancer and multikingdom microorganisms in cancer initiation and progression, and in responses to anticancer treatments.

## Abbreviations

CRC, colorectal cancer; IL, interleukin; PAMP, pathogen-associated molecular pattern; PRR, pattern recognition receptor.
